# Competition between surface modification and abrasive polishing: a method of controlling the surface atomic structure of 4H-SiC (0001)

**DOI:** 10.1038/srep08947

**Published:** 2015-03-10

**Authors:** Hui Deng, Katsuyoshi Endo, Kazuya Yamamura

**Affiliations:** 1 Research Center for Ultra-precision Science and Technology, Graduate School of Engineering, Osaka University, 2-1 Yamadaoka, Suita, Osaka 565-0871, Japan

## Abstract

The surface atomic step-terrace structure of 4H-SiC greatly affects its performance in power device applications. On the basis of the crystal structure of 4H-SiC, we propose the generation mechanism of the a-b-a*-b* type, a-b type and a-a type step-terrace structures. We demonstrate that the step-terrace structure of SiC can be controlled by adjusting the balance between chemical modification and physical removal in CeO_2_ slurry polishing. When chemical modification plays the main role in the polishing of SiC, the a-b-a*-b* type step-terrace structure can be generated. When the roles of physical removal and chemical modification have similar importance, the a-b-a*-b* type step-terrace structure changes to the a-b type. When physical removal is dominant, the uniform a-a type step-terrace structure can be generated.

Owing to the excellent electrical and mechanical properties of single-crystal silicon carbide (4H-SiC), it is considered to be one of the most attractive materials for high-power, high-frequency and high-temperature applications. However, because of its high hardness and chemical inertness, the flattening of 4H-SiC substrates is very difficult, which greatly limits the applicability of 4H-SiC. To fully utilize the excellent properties of 4H-SiC, such as its wide bandgap, high thermal conductivity and high breakdown electric field, the preparation of a SiC substrate with a “perfect” surface is essential. On a perfect SiC surface, no scratches or subsurface damaged layers should exist, and the surface should be atomically flat. In recent years, polishing techniques combining surface modification and mechanical polishing, such as chemical mechanical polishing^1^,^2^,^3^, catalyst-referred etching^4^,^5^,^6^,^7^, plasma-assisted polishing^8^,^9^,^10^,^11^ and so forth^12^,^13^, have been proposed for the damage-free and atomic-scale flattening of the Si (0001) face of 4H-SiC substrates. In all these techniques, the surface of the 4H-SiC substrate is modified by chemical reactions, and a modified layer that is easy to remove is generated. The modified layer is removed by abrasive polishing or etching to realize surface flattening. With the application of these polishing techniques, the generation of atomically flat SiC substrate surfaces with a well-ordered step-terrace structure has been reported.

Although the step-terrace structure obtained by these polishing techniques was well-ordered, a periodic step-terrace structure was usually formed. In plasma-assisted polishing, a step-terrace structure with four types of terrace that appear alternately is generated (a narrow terrace, a wide terrace and two terraces of intermediate width)^9^. This type of step-terrace structure is called the a-b-a*-b* type. In the case of catalyst-referred etching, a step-terrace structure with alternating narrow and wide terrace pairs has been reported^5^. We call this type of step-terrace structure the a-b type. In the case of SiC surfaces processed by chemical mechanical polishing, which is the most widely used method nowadays, an a-b type step-terrace structure or a step-terrace structure with a uniform terrace width, which is called the a-a type, can be observed on different substrates^14^.

Although the generation of the a-b-a*-b* type, a-b type and a-a type step-terrace structures on 4H-SiC has been reported in our previous research^11^, their generation mechanism has not been thoroughly clarified. These three types of step-terrace structure have occasionally been obtained in different polishing techniques^5^,^9^,^14^, and the process conditions under which they are generated are unclear. Therefore, the control of the step-terrace structure of 4H-SiC has not been realized. It is expected that control of the surface atomic structure of 4H-SiC will be highly advantageous for improving its performance in power device applications. In this study, the generation mechanism of these different types of step-terrace structure is proposed and experimentally clarified. Furthermore, control of the step-terrace structure of 4H-SiC was realized by adjusting the balance between chemical modification and physical removal in polishing.

## Results

### Crystal structure of 4H-SiC

**Figure 1:**
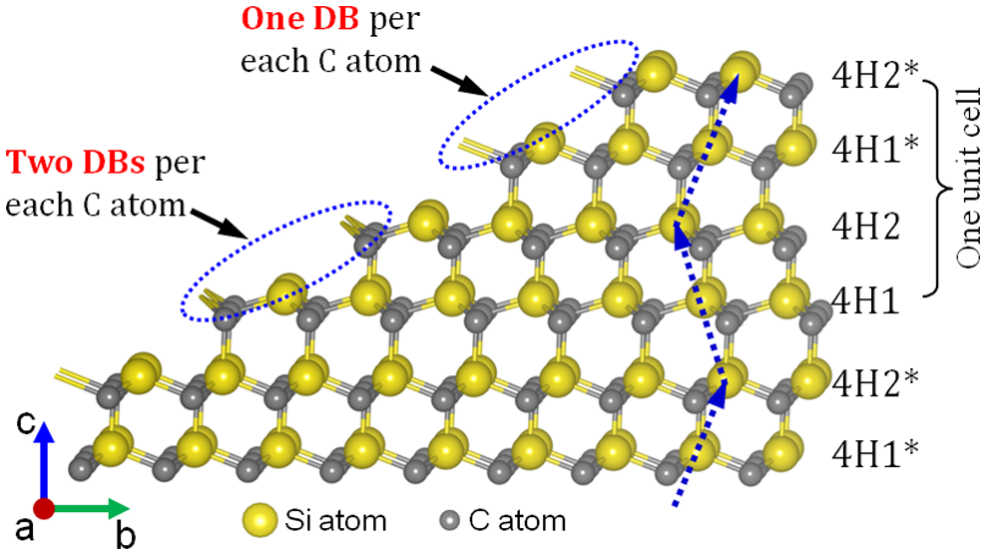
Crystal structure of 4H-SiC. Bond configuration of step-terrace structure on a 4H-SiC (0001) surface viewed from the [11–20] direction.

In all the above-mentioned polishing techniques, chemical modification, such as oxidation or etching, and physical removal, such as abrasive polishing, are combined^5^,^9^,^14^. Chemical modification softens the SiC substrate surface and abrasive polishing removes the modified layer to flatten the surface. Consistent with its name, there are four Si-C bilayers in one unit cell of 4H-SiC single crystal as shown in Figure 1. It is considered that there may be a relationship between the generation of these periodic step-terrace structures with different terrace widths and the four Si-C bilayers in a unit cell of 4H-SiC. In fact, it has been reported that there are two types of Si-C terrace, 4H1 and 4H2 terraces, existing in 4H-SiC depending on the physical relationship with the bilayers below^15^,^16^. According to a previous calculation result, the extra energy required to deposit a new layer on a 4H1 terrace is much higher than that for a 4H2 terrace^17^,^18^,^19^. In other words, a 4H1 terrace is much more stable than a 4H2 terrace. On the basis of this calculation result, Arima *et al*. proposed that the etching rates of 4H1 and 4H2 terraces are different, resulting in the generation of an a-b type step-terrace structure in a catalyst-referred etching process^20^. However, the difference between 4H1 and 4H2 terraces cannot explain the generation of a-b-a*-b* type and a-a type step-terrace structures. On the other hand, in the calculation of the extra energy required for deposition, the number of dangling bonds (DBs) at the step edge was not taken into consideration. When 4H-SiC is oxidized or polished, chemical reactions or physical removal starts from the step edge since the atoms at the step edge are the most unstable. Therefore, the number of DBs at the step edge strongly affects the oxidation rate (*r*_oxi_) of the terrace. As shown in Figure 1, taking the number of DBs of C atoms at the step edge into consideration, it is found that there are two terrace pairs in a unit cell of 4H-SiC. In one terrace pair, there is only one DB for each C atom at the step edge (4H1* and 4H2*), while in the neighboring terrace pair there are two DBs for each C atom at the step edge (4H1 and 4H2) as shown in Figure 1. This means that *r*_oxi_ for the 4H1-4H2 terrace pair is higher than that for the 4H1*-4H2* terrace pair. Also, in the terrace pairs 4H1-4H2 and 4H1*-4H2*, *r*_oxi_ for 4H2 or 4H2* is higher than that for 4H1 or 4H1* according to the results of previous first-principles calculations^15^,^16^. Correspondingly, it is concluded that these four types of Si-C terrace in a unit cell of 4H-SiC (4H1, 4H2, 4H1* and 4H2*) have different values of *r*_oxi_.

### Generation mechanism of different step-terrace structures

When 4H-SiC is oxidized, oxidation starts from the step edges, which are the most unstable areas on the substrate surface. Since there are four values of *r*_oxi_ in a unit cell of 4H-SiC, the widths of the corresponding oxidized terraces are different. Therefore, it is reasonable to consider that the generation of the a-b-a*-b* type step-terrace structure results from the four types of Si-C terrace, with different values of *r*_oxi_. However, the a-b type and a-a type step-terrace structures can also be observed in other polishing techniques. Obviously the generation of these two types of step-terrace structure cannot be explained only by the oxidation process.

In the above-mentioned polishing techniques, chemical reactions and abrasive polishing simultaneously occur^5^,^9^,^14^. Since the values of *r*_oxi_ for the four types of Si-C terrace in a unit cell are different, the oxidation process preferentially leads to different step widths in a unit cell of 4H-SiC, resulting an a-b-a*-b* type step-terrace structure. On the other hand, in the abrasive polishing process, the modified layer is first removed. Then, there is abrasive contact with SiC at the step edge and strain is introduced. With the repetition of this process, Si-C terraces are removed from the step edge. Since abrasive polishing is a physical removal process, the removal rates by polishing (*r*_pol_) of the four types of Si-C terrace in 4H-SiC are the same. This means that abrasive polishing preferentially leads to a uniform step width, resulting in an a-a type step-terrace structure. In the above polishing techniques, both chemical modification and physical removal occur. It is considered that under different polishing conditions, the balance between chemical modification and physical removal is different, which leads to different types of step-terrace structure.

**Figure 2:**
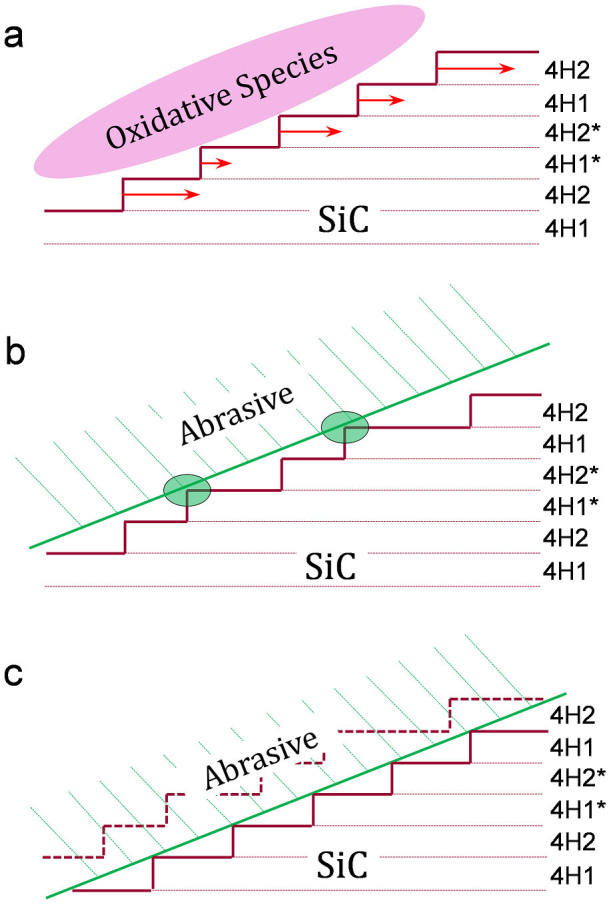
Generation mechanism of step-terrace structure of 4H-SiC. (a) Surface modification was dominant, resulting in the generation of the a-b-a*-b* type step-terrace structure. (b) Physical removal was comparable with surface modification, resulting in the generation of the a-b type step-terrace structure. (c) Physical removal was dominant, resulting in the generation of the a-a type step-terrace structure.

On the basis of the above analysis, we consider that the generation of the three types of step-terrace structures, a-a type, a-b type, and a-b-a*-b* type, can be controlled by changing the balance between chemical modification and physical removal in the polishing of 4H-SiC. The generation mechanism of the three types of step-terrace structure of 4H-SiC is proposed as shown in Figure 2. This mechanism is based on the balance between *r*_oxi_ and *r*_pol_. As shown in Figure 2a, in the case that chemical modification plays the main role in slurry polishing, *r*_oxi_ is higher than *r*_pol_ and abrasive polishing only removes the modified oxide layer. As previously introduced, the four types of Si-C terrace in a unit cell of 4H-SiC, 4H1, 4H2, 4H1* and 4H2*, have different values of *r*_oxi_. The oxidation of SiC starts from the step edge. For these four types of Si-C terrace, the widths of the corresponding oxidized terraces are different. After a modified layer is generated, it is removed by abrasive polishing. Upon the removal of the modified layers, the a-b-a*-b* type step-terrace structure is generated.

If the value of *r*_pol_ for abrasive polishing increases and become comparable with *r*_oxi_ for surface modification, as shown in Figure 2b, contact between the abrasive and the SiC terraces occurs. The diameter of the abrasive particles is very large compared with the height of the SiC steps of 0.25 nm. Thus, after the a-b-a*-b* type step-terrace structure is generated, the step edge of the wide terraces preferentially comes in contact with the abrasive particles. If *r*_pol_ is low, the amount of physical removal is very limited and the modified layer is mainly removed. However, contact between the abrasive and the wide terraces frequently occurs when the value of *r*_pol_ for abrasive polishing increases. Owing to the contact between the abrasive and SiC, strain is applied to the contact area. The area subjected to strain is rapidly oxidized and immediately removed by abrasive polishing, i.e., the wide terraces are preferentially removed. Thus, the a-b-a*-b* type step-terrace structure is changed to the a-b type. In this case, the physical removal factor is comparable to the chemical oxidation factor in the polishing of SiC.

Finally, if the value of *r*_pol_ for abrasive polishing is greatly increased to higher than the values of *r*_oxi_ for Si-C terraces, physical removal plays the main role as shown in Figure 2c. In the case of a high *r*_pol_ for abrasive polishing, the modified layer is removed rapidly and physical removal of the Si-C terraces occurs. Owing to the high *r*_pol_, all the terraces in SiC are in uniform contact with the abrasive particles after the oxide layer is removed. Therefore, all the terraces are removed at the same rate and the uniform a-a type step-terrace structure is generated.

### Control of the step-terrace structure

To prove the validity of the proposed mechanism, 4H-SiC (0001) substrates were polished using CeO_2_ slurry with different rotation speeds of the polishing pad. CeO_2_ slurry was used because it has been widely reported that some Si-based materials, such as Si, SiO_2_, Si_3_N_4_ and SiC, can be polished by CeO_2_ abrasive owing to its tribocatalytic properties^21^,^22^,^23^,^24^,^25^. In the polishing of 4H-SiC substrates with CeO_2_ slurry, modification of the SiC substrate surface and abrasive polishing to remove the modified layer are combined. The surface of SiC is modified to silicon oxycarbide (Si-C-O) and further modified to SiO_2_ owing to the tribocatalytic effect of the CeO_2_ abrasive^26^. Also, this modification process is promoted by the chemicals in the slurry such as hydroxide (OH) as well as the lattice strain caused by the pressure applied by the polishing pad^27^. After the surface is modified, the modified layer is immediately removed by the CeO_2_ abrasive. The composition of the slurry and the load during polishing affect the value of *r*_oxi_ for SiC in CeO_2_ slurry polishing. Therefore, SiC is polished under a constant load with the same slurry, which means that the factors affecting the chemical modification of SiC do not change in the polishing experiments. The pad rotation speed is increased in three stages from 500 rpm to 2500 rpm. According to Preston's law, the value of *r*_pol_ in abrasive polishing increases linearly with the relative speed between the specimen and the polishing pad. This means that the value of *r*_pol_ in abrasive polishing increases with the pad rotation speed^28^. In this way, the balance between the chemical modification (*r*_oxi_) and physical removal (*r*_pol_) of SiC in CeO_2_ slurry polishing can be controlled.

**Figure 3:**
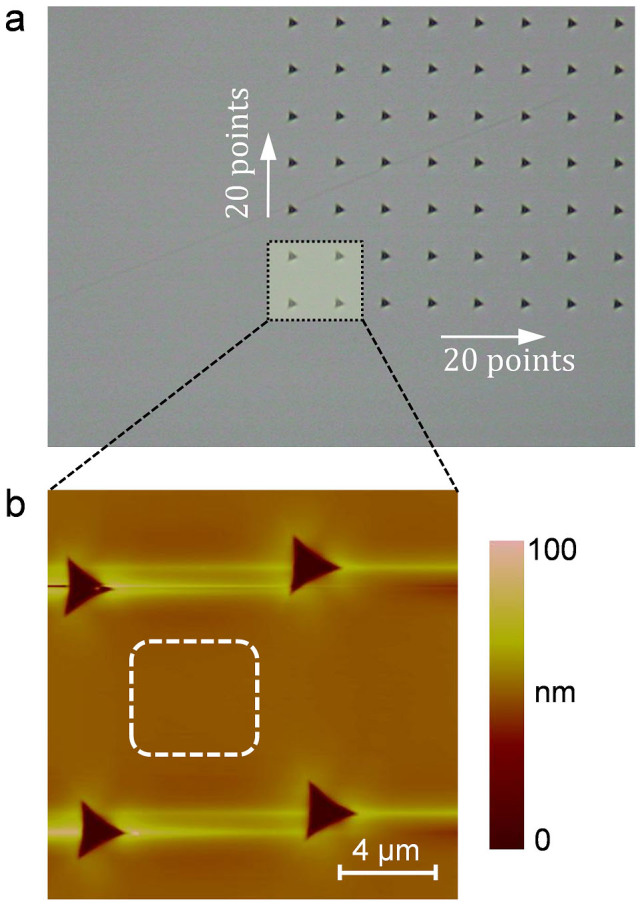
Indents on the SiC surface. (a) CCD image of the indents formed on the surface of 4H-SiC to be polished. (b) AFM image of the lower-left corner of the indented area. The change in the step-terrace structure on the polished surface after each polishing experiment was observed in the same area indicated by the dashed square.

A SiC specimen was first polished by CeO_2_ slurry with a pad rotation speed of 500 rpm. Then, the same specimen was polished by CeO_2_ slurry with the pad rotation speed increased to 1500 rpm followed by polishing with a pad rotation speed of 2500 rpm. To confirm the change in the step-terrace structure with the increase in the pad rotation speed, it is very helpful to observe the same area of the same specimen. However, owing to the small observation area in atomic force microscopy (AFM), it is very difficult to find the same place on the same specimen after each polishing experiment. Therefore, a nanoindenter was used to form some indents on the surface to be polished, which were helpful for finding the same area in AFM observation. Figure 3a shows an image of the indents obtained by the charge-coupled device (CCD) camera of the microscope. Using these indents as a reference, the AFM observation after each polishing experiment was localized to the area in the lower-left corner, which included four indents as shown in Figure 3b. In this way, the change in the step-terrace structure could be observed at the same area of the same specimen.

**Figure 4:**
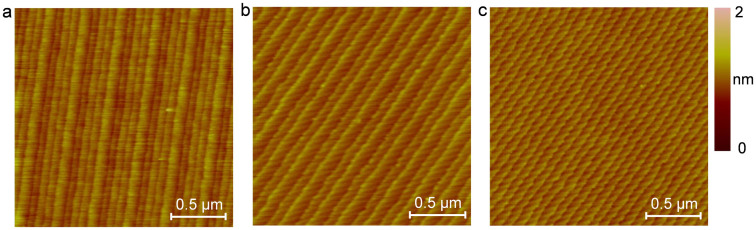
AFM observation of polished SiC surfaces. Change in the step-terrace structure with increasing pad rotation speed observed by AFM. (a) 500 rpm. (b) 1500 rpm. (c) 2500 rpm.

The change in the step-terrace structure with the increase in the pad rotation speed is shown in Figure 4. When the surface was polished with a pad rotation speed of 500 rpm, the a-b-a*-b* type step-terrace structure was generated as shown in Figure 4a. In such a step-terrace structure, four types of terrace, a narrow terrace, a wide terrace and two terraces with intermediate width, alternately appeared on the polished surface. After this specimen was polished with a pad rotation speed of 1500 rpm, the step-terrace structure changed from the a-b-a*-b* type to the a-b type as shown in Figure 4b. In the a-b type step-terrace structure, terrace pairs with narrow and wide terraces alternately appeared on the polished surface. Finally, after the specimen was polished with a pad rotation speed of 2500 rpm, the step-terrace structure changed from the a-b type to the uniform a-a type, as shown in Figure 4c, in which all the terraces had the same width.

It was concluded that the step-terrace structure on the polished SiC substrate surface could be changed by changing the pad rotation speed. When the pad rotation speed increased from 500 rpm to 1500 rpm and 2500 rpm, the step-terrace structure changed from the a-b-a*-b* type to the a-b type and uniform a-a type, respectively. Consistent with the mechanism proposed in Figure 2, increasing the pad rotation speed increased the value of *r*_pol_ for abrasive polishing, resulting in a change in the balance between surface modification and abrasive polishing in slurry polishing.

**Figure 5:**
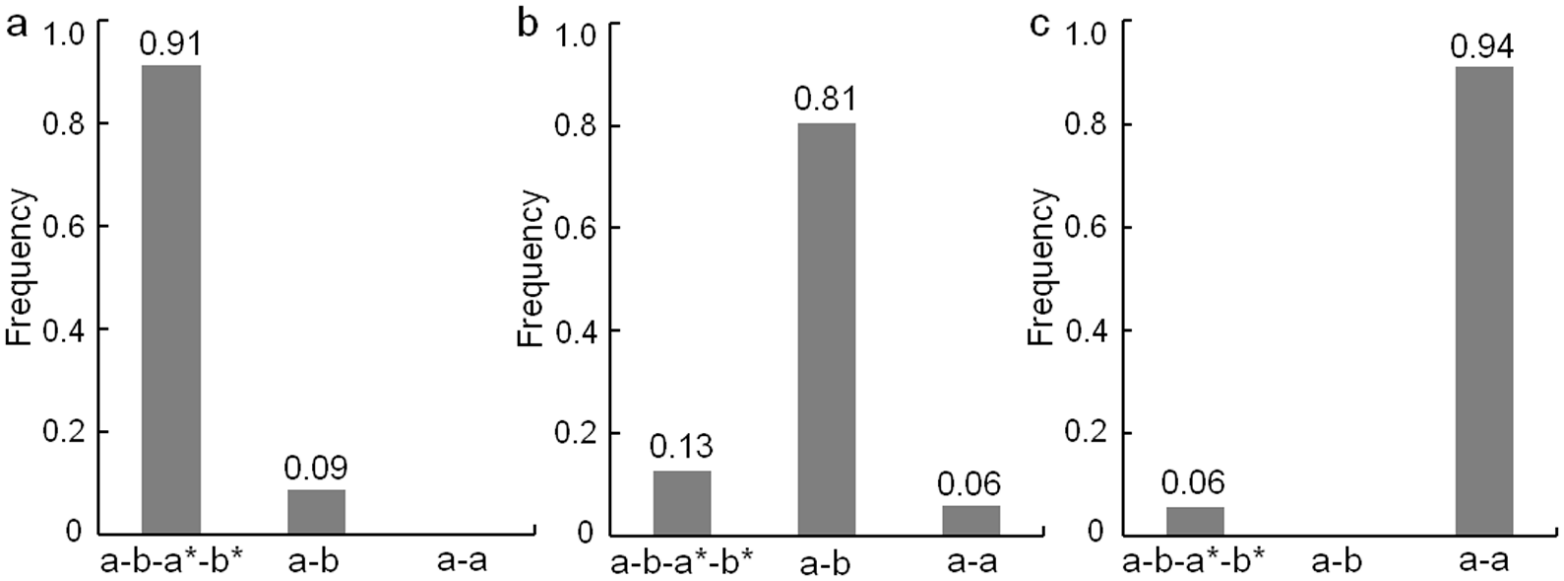
Step-terrace structure distribution. Distribution of the a-b-a*-b* type, a-b type and a-a type step-terrace structures on 4H-SiC substrate surfaces polished with different pad rotation speeds. (a) 500 rpm. (b) 1500 rpm. (c) 2500 rpm.

According to the results of AFM observation, the change in the step-terrace structure in the same area was confirmed. However, compared with the AFM observation area, the polished area was much larger. Therefore, it was necessary to evaluate the distribution of step-terrace structures on the whole polished area after each polishing experiment. For each specimen, 100 points uniformly distributed over the polished area were observed. Figure 5 shows the distribution of different types of step-terrace structure on 4H-SiC substrate surfaces polished with different pad rotation speeds. In the case of a pad rotation speed of 500 rpm, although both the a-b-a*-b* type and a-b type step-terrace structures were observed, the a-b-a*-b* type had a very high frequency of 91% as shown in Figure 5a. Also, in this case, the a-a type step-terrace structure was not found. On the surface polished with a pad rotation speed of 1500 rpm, as shown in Figure 5b, all three types of step-terrace structure were observed and the a-b type step-terrace structure had a high frequency of 81%. When the pad rotation speed was increased to 2500 rpm, 94% of the observed areas showed the a-a type step-terrace structure.

### Surface composition after polishing

**Figure 6:**
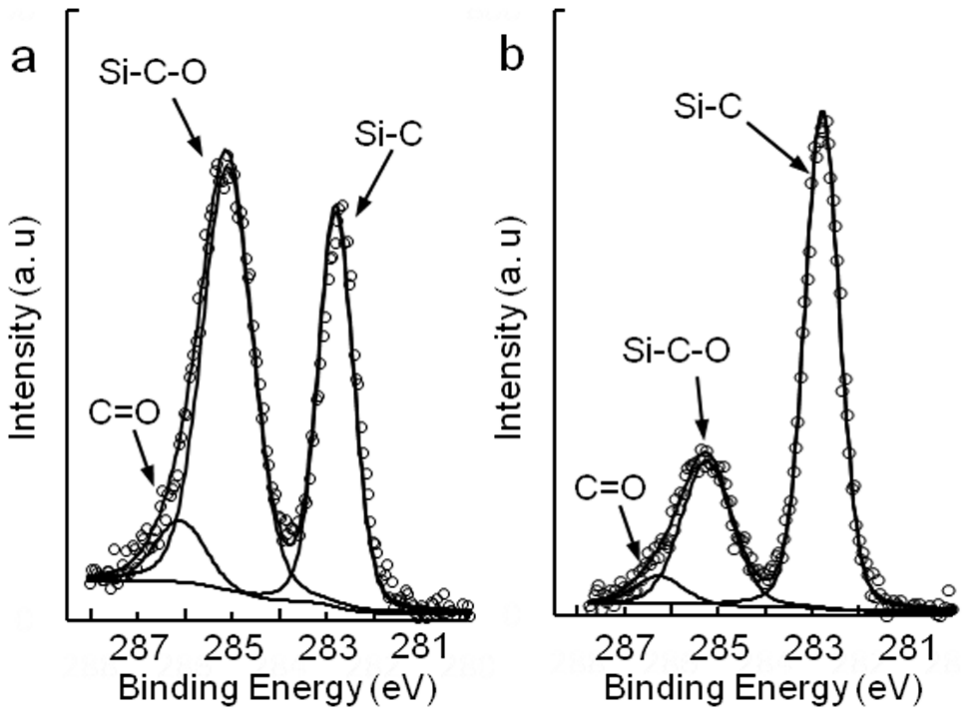
Residual oxidation products on the polished surfaces. C1s spectra of the SiC substrate surfaces polished with pad rotation speeds of (a) 500 rpm and (b) 2500 rpm.

According the proposed mechanism, with the change in the balance between chemical modification and physical removal caused by changing the pad rotation speed, there should be more oxidation products on a surface polished with a low pad rotation speed than on a surface polished with a high pad rotation speed. Therefore, the residual oxidation products on the polished surfaces were determined by angle-resolved X-ray photoelectron spectroscopy (ARXPS). To increase the difference in the amount of residual oxidation products on the polished surfaces, SiC surfaces polished with the lower pad rotation speed of 500 rpm and the higher pad rotation speed of 2500 rpm were used for ARXPS measurements. Figure 6 shows the carbon core-level (C1s) spectra of the SiC substrate surfaces polished by CeO_2_ slurry with pad rotation speeds of 500 rpm and 2500 rpm. The strong Si-C peaks were assigned to the bulk SiC. Other peaks, such as Si-C-O and C = O, were considered to originate from residual oxidation products on the polished surfaces^29^,^30^. On the surface polished with a pad rotation speed of 500 rpm, it was found that the Si-C-O peak intensity was even stronger than that of Si-C as shown in Figure 6a; thus, the thickness of silicon oxycarbide was greater than that of SiC within the detection depth of XPS. Figure 6b shows the C1s spectra of the surface polished with a pad rotation speed of 2500 rpm. The amount of oxidation products on this surface was small since the intensities of the Si-C-O and C = O peaks were relatively weak compared with those of the surface polished at 500 rpm. According to our proposed mechanism, for a pad rotation speed of 500 rpm, chemical reactions played the main role in CeO_2_ slurry polishing; thus, there should be more oxidation products remaining on the polished surface. On the other hand, for a pad rotation speed of 2500 rpm, *r*_pol_ was increased and physical removal played the main role, therefore the amount of residual oxidation products should be small. The results of XPS measurement shown in Figure 6 support our proposed mechanism.

## Discussion

In our previous research, the a-b-a*-b* type step-terrace structure was obtained by plasma-assisted polishing^8^,^9^,^10^. In plasma-assisted polishing, the irradiation of atmospheric-pressure water vapor plasma was used for surface modification. Plasma irradiation and abrasive polishing (CeO_2_) were simultaneously conducted for surface flattening. For the oxidation of 4H-SiC, water vapor plasma has a very high initial *r*_oxi_ of 0.185 μm/h^31^. The value of *r*_pol_ for abrasive polishing was much lower than that of *r*_oxi_ for oxidation in water vapor plasma. According to the proposed mechanism, the high *r*_oxi_ was the reason for the generation of the a-b-a*-b* type step-terrace structure in plasma-assisted polishing.

In summary, the generation of the a-b-a*-b* type, a-b type and a-a type step-terrace structures of 4H-SiC could be controlled by adjusting the balance between chemical modification and physical removal. When *r*_pol_ for abrasive polishing was lower than the rate of surface modification, the a-b-a*-b* type step-terrace structure was generated. When the pad rotation speed was increased and *r*_pol_ for abrasive polishing became comparable to the rate of surface modification, the step-terrace structure changed from the a-b-a*-b* type to the a-b type. When *r*_pol_ for abrasive polishing was higher than the rate of surface modification, a uniform a-a type step-terrace structure was generated. On the basis of this mechanism, the results of polishing using existing polishing techniques in which chemical reactions and abrasive polishing are combined can be explained. Furthermore, it is expected that the control of the step-terrace structure on a 4H-SiC substrate will be very advantageous for realizing excellent device performance, which will be experimentally confirmed in future research.

## Methods

### CeO_2_ slurry polishing

Commercially available single-crystal 4H-SiC substrates (on-axis, n-type) supplied by TanKeBlue Semiconductor Co. Ltd. were used in this work. All the experiments were conducted on the Si (0001) face, which is the most commonly used face for power devices. Polishing pads (NP178) with a diameter of 10 mm supplied by FILWEL Co. Ltd. were used. The concentration of CeO_2_ slurry was 1 wt% and its pH was 8.53. The average diameter of the CeO_2_ abrasive particles in the slurry was 190 nm. Both the polishing pad and the SiC specimen were immersed in CeO_2_ slurry. The polishing pad was scanned on the specimen with a scanning speed of 200 mm/min. The polishing pressure was 3.743 kPa. The pad rotation speed was increased from 500 rpm to 1500 rpm and finally 2500 rpm. Each specimen was polished for 3 h. In each polishing experiment, a new polishing pad was used and the slurry was replaced.

### Surface characterization

A nanoindenter (ENT-2100, ELIONIX Inc.) was used to form some indents on the specimen, which were used as a reference in AFM observations. The maximum load applied for indentation was 100 mN. A total of 400 indents (20 × 20) separated by a distance of 10 μm were formed. The surface morphology of the polished SiC substrates was measured by AFM (SPA-400, SII Nanotechnology) in the tapping mode. For each specimen, 100 points that were uniformly distributed over the polished area were observed. The surface composition of the SiC substrates polished with pad rotation speeds of 500 rpm and 2500 rpm was determined by XPS (Quantum 2000, ULVAC-PHI) with AlKα radiation (1486.6 eV). To centralize the XPS observation to the top surface, the stage on which the specimen was located was tilted with a very low takeoff angle of 10°. Before the XPS measurements, to remove the organic contaminants on the specimen, cleaning in a sulfuric acid (H_2_SO_4_) and hydrogen peroxide (H_2_O_2_) mixture (SPM) was conducted for 10 min followed by cleaning in pure water for 10 min. The concentration of the SPM solution was H_2_SO_4_ (97 wt%): H_2_O_2_ (30 wt%) = 4:1. For each specimen, five points uniformly distributed over the polished area were observed to exclude any area dependence.

## Author Contributions

H.D. and K.Y. conceived this research. H.D. performed the experiments, analyzed the data and wrote the manuscript. K.Y. supervised H.D. and edited the manuscript. K.E. participated in discussion through the work.

## References

[b1] Zhou L., Audurier V., Pirouz P. & Powell J. A. Chemomechanical polishing of silicon carbide. J. Electrochem. Soc. 144, L161–L163 (1997).

[b2] Neslen C. L., Mitchel W. C. & Hengehold R. L. Effects of process parameter variations on the removal rate in chemical mechanical polishing of 4H-SiC. J. Electron. Mater. 30, 1271–1275 (2001).

[b3] Aida H. *et al.* Ultraprecision CMP for sapphire, GaN, and SiC for advanced optoelectronics Materials. Curr. Appl. Phys. 12, S41–S46 (2012).

[b4] Hara H. *et al.* Novel abrasive-free planarization of 4H-SiC (0001) using catalyst. J. Electron. Mater. 35, L11–L14 (2006).

[b5] Okamoto T. *et al.* Dependence of process characteristics on atomic-step density in catalyst-referred etching of 4H–SiC (0001) surface. J. Nanosci. Nanotech. 11, 2928–2930 (2011). 10.1166/jnn.2011.3917 21776655

[b6] Okamoto T. *et al.* Improvement of removal rate in abrasive-free planarization of 4H-SiC substrates using catalytic platinum and hydrofluoric acid. Jpn. J. Appl. Phys. 51, 046501 (2012).

[b7] Sano Y., Arima K. & Yamauchi K. Planarization of SiC and GaN wafers using polishing technique utilizing catalyst surface reaction. ECS J. Solid State Sci. Tech. 2, N3028–N3035 (2013).

[b8] Yamamura K. *et al.* Plasma assisted polishing of single crystal SiC for obtaining atomically flat strain-free surface. CIRP Ann. 60, 571–574 (2011).

[b9] Deng H., Ueda M. & Yamamura K. Characterization of 4H-SiC (0001) surface processed by plasma-assisted polishing. Int. J. Adv. Manuf. Technol. 72, 1–7 (2014).

[b10] Deng H. & Yamamura K. Atomic-scale flattening mechanism of 4H-SiC (0001) in plasma assisted polishing. CIRP Ann. 62, 575–578 (2013).

[b11] Deng H., Monna K., Tabata T., Endo K. & Yamamura K. Optimization of the plasma oxidation and abrasive polishing processes in plasma-assisted polishing for highly effective planarization of 4H-SiC. CIRP Ann. 63, 529–532 (2014).

[b12] Kubota A., Yoshimura M., Fukuyama S., Iwamoto C. & Touge M. Planarization of C-face 4H-SiC substrate using Fe particles and hydrogen peroxide solution. Precis. Eng. 36, 137–140 (2012).

[b13] Kikuchi M., Takahashi Y., Suga T., Suzuki S. & Bando Y. Mechanochemical Polishing of silicon carbide Single crystal with chromium (111) oxide abrasive. J. Am. Ceram. Soc. 75, 189–194 (1992).

[b14] Shi X. L., Pan G. S., Zhou Y., Zou C. L. & Gong H. Extended study of the atomic step-terrace structure on hexagonal SiC (0001) by chemical-mechanical planarization. Appl. Surf. Sci. 284, 195–206 (2013).

[b15] Shaw J. J. A. & Heine V. The nature of interplanar interactions in SiC polytypes. J. Phys.: Condens. Mater. 2, 4351–4361 (1990).

[b16] Chien F. R., Nutt S. R., Yoo W. S., Kimoto T. & Matsunami H. Terrace growth and poly type development in epitaxial β-SiC films on α-SiC (6H and 15R) substrates. J. Mater. Res. 9, 940–954 (1994).

[b17] Kimoto T., Itoh A., Matsunami H. & Okano T. Step bunching mechanism in chemical vapor deposition of 6H– and 4H–SiC{0001}. J. Appl. Phys. 81, 3494–3500 (1997).

[b18] Heine V., Cheng C. & Needs R. J. The Preference of Silicon Carbide for Growth in the Metastable Cubic Form. J. Am. Ceram. Soc. 74, 2630–2633 (1991).

[b19] Yazdi G. R. *et al.* Growth of large area monolayer graphene on 3C-SiC and a comparison with other SiC polytypes. Carbon 57, 477–484 (2013).

[b20] Arima K. *et al.* Atomic-scale flattening of SiC surfaces by electroless chemical etching in HF solution with Pt catalyst. Appl. Phys. Lett. 90, 202106 (2007).

[b21] Hoshino T., Kurata Y., Terasaki Y. & Susa K. Mechanism of polishing of SiO_2_ films by CeO_2_ particles. J. Non-Cryst Solids 283, 129–136 (2001).

[b22] Oh M. H., Singh R. K., Gupta S. & Cho S. B. Polishing behaviors of single crystalline ceria abrasives on silicon dioxide and silicon nitride CMP. Microelectron. Eng. 87, 2633–2637 (2010).

[b23] Zho L. *et al.* Defect-free fabrication for single crystal silicon substrate by chemo-mechanical grinding. CIRP Ann. 55, 313–316 (2006).

[b24] Tian Y. B., Zhou L., Shimizu J., Tashiro Y. & Kang R. K. Elimination of surface scratch/texture on the surface of single crystal Si substrate in chemo-mechanical grinding (CMG) process. Appl. Surf. Sci. 255, 4205–4211 (2009).

[b25] Kamiya S. *et al.* Study on reaction mechanism of Si and pure CeO_2_ for chemical-mechanical-grinding Process. J. Vac. Sci. Technol. B 27, 1496–1502 (2009).

[b26] Kido T., Nagaya M., Kawata K. & Kato T. A novel grinding technique for 4H-SiC single-crystal wafers using tribo-catalytic abrasives. Mater. Sci. Forum 778–780, 754–758 (2014).

[b27] Rajendran A., Takahashi Y., Koyama M., Kubo M. & Miyamoto A. Tight-binding quantum chemical molecular dynamics simulation of mechano-chemical reactions during chemical–mechanical polishing process of SiO_2_ surface by CeO_2_ particle. Appl. Surf. Sci. 244, 34–38 (2005).

[b28] Preston F. W. The theory and design of Plate glass polishing machines. J. Soc. Glass Technol. 11, 214–256 (1927).

[b29] Palmieri R., Radtke C., Boudinov H. & Silva E. F. Improvement of SiO_2_/4H-SiC interface properties by oxidation using hydrogen Peroxide. Appl. Phys. Lett. 95, 113504 (2009).

[b30] Knaup J. M. *et al.* Theoretical study of the mechanism of dry oxidation of 4H-SiC. Phys. Rev. B 71, 235321 (2005).

[b31] Deng H., Endo K. & Yamamura K. Comparison of thermal oxidation and plasma oxidation of 4H-SiC (0001) for surface Flattening. Appl. Phys. Lett. 104, 101608 (2014).

